# Magnetic Separation and Antibiotics Selection Enable Enrichment of Cells with ZFN/TALEN-Induced Mutations

**DOI:** 10.1371/journal.pone.0056476

**Published:** 2013-02-18

**Authors:** Hyojin Kim, Myung-Sun Kim, Gabbine Wee, Choong-il Lee, Hyongbum Kim, Jin-Soo Kim

**Affiliations:** 1 National Creative Research Initiatives Center for Genome Engineering and Department of Chemistry, Seoul National University, Seoul, South Korea; 2 Graduate School of Biomedical Science and Engineering/College of Medicine, Hanyang University, Seoul, Korea; Center for Genomic Regulation, Spain

## Abstract

The ability to enrich cells with targeted mutations greatly facilitates the process of using engineered nucleases, including zinc-finger nucleases and transcription activator-like effector nucleases, to construct such cells. We previously used surrogate reporters to enrich cells containing nuclease-induced mutations via flow cytometry. This method is, however, limited by the availability of flow cytometers. Furthermore, sorted cells occasionally fail to form colonies after exposure to a strong laser and hydrostatic pressure. Here we describe two different types of novel reporters that enable mutant cell enrichment without the use of flow cytometers. We designed reporters that express H-2K^k^, a surface antigen, and the hygromycin resistance protein (Hygro^R^), respectively, when insertions or deletions are generated at the target sequences by the activity of engineered nucleases. After cotransfection of these reporters and the engineered nuclease-encoding plasmids, H-2K^k^- and Hygro^R^-expressing cells were isolated using magnetic separation and hygromycin treatment, respectively. We found that mutant cells were drastically enriched in the isolated cells, suggesting that these two reporters enable efficient enrichment of mutants. We propose that these two reporters will greatly facilitate the use of engineered nucleases in a wider range of biomedical research.

## Introduction

Engineered nucleases, including zinc-finger nucleases (ZFNs) and TAL-effector nucleases (TALENs), are promising tools for targeted genetic engineering [1]. The ability to enrich cells with targeted mutations greatly facilitates the process of using engineered nucleases to construct such cells [2]. We previously developed surrogate reporters that enable the efficient enrichment of cells containing nuclease-induced mutations via flow cytometry [3]. This method is, however, limited by the availability of flow cytometers. Furthermore, sorted cells occasionally fail to form colonies after exposure to a strong laser and hydrostatic pressure. Thus, we attempted to develop methods to select mutant cells without the use of flow cytometers.

Magnetic separation has been used as an alternative method to isolate cells that express specific antigens [4], [5]. Magnetic separation does not require flow cytometers and is faster and easier to perform than flow cytometric sorting [4], [6]. To separate transgenic cells from wild-type cells immunomagnetically, H-2K^k^, a truncated mouse MHC class I molecule, is used as a selection marker [7], [8]. H-2K^k^ is expressed only in some rare mouse strains such as AKR/J or CBA/J, but not in human or most other murine cells [9], [10], rendering H-2K^k^ a good marker to distinguish transgenic cells from control cells. To avoid any effects generated by the expression of H-2K^k^, a truncated H-2K^k^ that lacks a cytoplasmic domain is used [7], [8]. Magnetic separation using H-2K^k^ is effective in the enrichment of transiently transfected cells [11] and lenti- or retro-virally transduced cells [8], [12]. Here we adopt this system to enrich mutant cells generated by engineered nucleases.

Selection of cells using resistance factors against antibiotics is widely used for the isolation of genetically-modified cells in prokaryotes [13], [14] and eukaryotes [15], [16]. Hygromycin B is an aminoglycoside antibiotic produced by the bacterium *Stepretomyces hygroscopicus*, which kills both prokaryotes and eukaryotes by inhibiting protein synthesis through interference with aminoacyl-tRNA recognition and ribosomal translocation [16]–[18]. Hygromycin B phosphotransferase, encoded by the hygromycin-resistance gene that was originally isolated from *Escherichia coli*, phosphorylates hygromycin B, resulting in its inactivation [14]. This gene has been successfully used as a selection marker for transformed prokaryotes [19] and transgenic eukaryotes [15], [16]. The hygromycin resistance gene has also been adopted to prepare donor DNA that will be integrated into a host genome via engineered nuclease-enhanced homologous recombination, allowing selection of cells with targeted genetic modifications [20], [21]. However, the isolation of engineered nuclease-induced mutant cells using hygromycin selection based on transiently active episomal reporters has yet to be demonstrated.

Here we present two novel reporter systems that enable enrichment of nuclease-induced mutant cells using magnetic separation and hygromycin selection. These reporters express H-2K^k^ and the hygromycin resistance protein, respectively, only when insertions or deletions (indels) are generated at the target sequences in the reporter systems, enabling efficient enrichment of mutant cells without using a flow cytometer.

## Materials and Methods

### Reporter vector construction

The 2A sequence was inserted into the pRGS reporter [3] using synthesized oligonucleotides (Bioneer, Daejon, South Korea). The mouse H2-K^k^ gene was amplified from pMACS K^k^ (Miltenyi Biotech, Germany) using appropriate primers (Table S1), and the amplified product was cloned into the modified pRGS vector by isothermal cloning [22]. The hygromycin B resistance gene was amplified from pBABE-hygro-hTERT (Addgene, plasmid #1773) using appropriate primers (Table S1), and the amplified product was cloned into the NheI site of the modified pRGS vector.

### ZFNs, TALENs, and reporters

Plasmids encoding the ZFNs and TALENs used in this study were previously described [3], [23] or obtained from ToolGen (Seoul, South Korea). Reporters were prepared as previously described [3]. Briefly, oligonucleotides that contained target sites were synthesized (Bioneer, Daejon, South Korea) and annealed in vitro. The annealed oligonucleotides were ligated into the vector. The sequences of reporters that contain Z891 target sites are included in Notes S1 and S2.

### Cell culture

Human embryonic kidney 293T (HEK293T) cells and Huh 7.5 cells were maintained in Dulbecco's modified Eagle medium (DMEM, Invitrogen) supplemented with 100 units/ml penicillin, 100 µg/ml streptomycin, and 10% fetal bovine serum.

### Transfection

Cells were transfected using lipofectamine 2000 (Invitrogen, Carlsbad, CA) or polyethyleneimine (linear, MW∼25,000, Polysciences) at a weight ratio of 1∶1∶2 (plasmid encoding a ZFN: plasmid encoding the other ZFN: magnetic reporter) or 10∶10∶1 (hygromycin reporter). Huh 7.5 cells were electroporated using a 100-µl tip at voltage 1, 100 V, width 30 ms, and one pulse in the Neon Transfection System (Invitrogen) with a total of 8 µg plasmid DNA (1∶1∶2 weight ratio).

### Magnetic separation

The transfected cells were cultured for one day at 37°C followed by culture at 30°C (cold shock) [24] for two days and subjected to magnetic separation. Trypsinized cell suspensions were mixed with magnetic bead-conjugated antibody against H-2K^k^ (MACSelect K^k^ microbeads; Miltenyi Biotech, Germany) and incubated for 20 min at 4°C. Labeled cells were separated using a column (MACS LS column; Miltenyi Biotech) according to the manufacturer's instructions.

### Hygromycin selection

Two days after transfection, hygromycin selection was performed by culturing the cells in the presence of 2 mg/ml of hygromycin B for two days at 37°C. For clonal analysis, hygromycin-selected cells were plated at a density of 3,000 cells/100 mm dish, and the clonal colonies were manually picked 10 days after plating.

### T7E1 assay

The T7E1 assay was performed as previously described [3], [23]. Briefly, genomic DNA was isolated using the DNeasy Blood & Tissue Kit (Qiagen) according to the manufacturer's instructions. The region of DNA containing the nuclease target site was PCR-amplified using the primers previously described [3]. The amplicons were denatured by heating and annealed to form heteroduplex DNA, which was treated with 5 units of T7 endonuclease 1 (New England Biolabs) for 15 to 20 min at 37°C and then analyzed by 2% agarose gel electrophoresis.

### Sequencing analysis

PCR amplicons that included ZFN-target sites were purified using the Gel Extraction Kit (MACHERRY-NALGEN) and cloned into the T-Blunt vector using the T-Blunt PCR Cloning Kit (SolGent). Cloned plasmids were sequenced using the primers used for PCR amplification.

## Results and Discussion

### Enrichment of mutant cells using magnetic reporters

**Figure 1:**
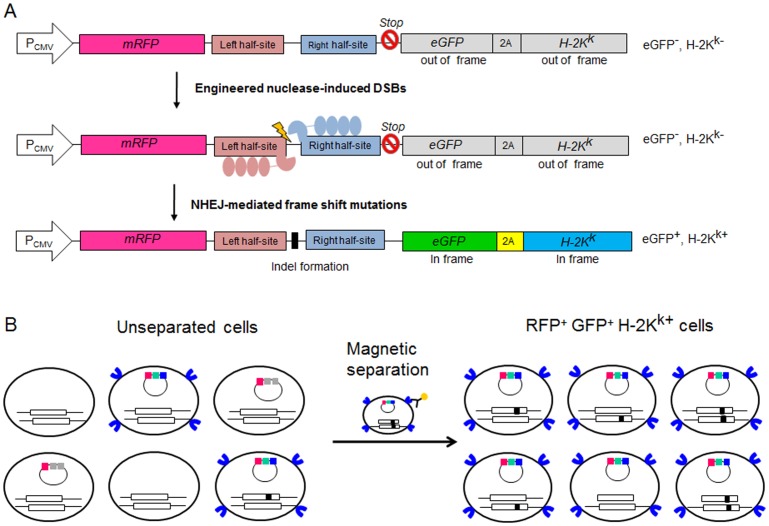
Overview of the episomal reporters used for the enrichment of nuclease-induced mutant cells via magnetic separation. (A) The working mechanism of the H-2K^k^ magnetic reporter. mRFP is constitutively expressed by the CMV promoter (P_CMV_), whereas eGFP and H-2K^k^ are not expressed without the activity of engineered nucleases because their sequences are out of frame. If a double-strand break is introduced into the target sequence by engineered nucleases, the break is repaired by nonhomologous end-joining (NHEJ), which often results in indels. Indel generation can cause frame shifts, making eGFP and H-2K^k^ in frame and leading to the expression of eGFP and H-2K^k^. (B) A schematic depicting the enrichment of mutant cells using the H-2K^k^ reporter. H-2K^k^-expressing cells can be magnetically separated using anti-H-2k^k^ antibody conjugated to magnetic beads. Mutant cells were enriched in this population of H-2k^k^-expressing cells. Reporter plasmids and chromosomal target loci are shown. Black spots represent mutations.

**Figure 2:**
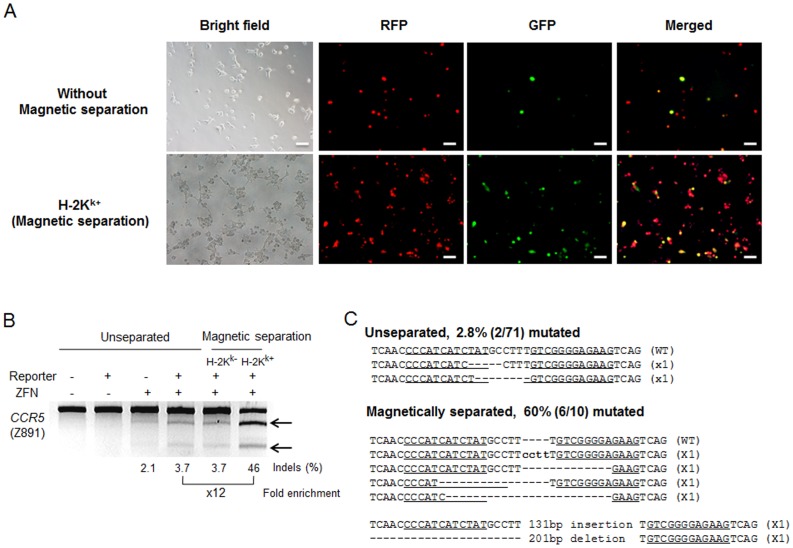
Magnetic separation-mediated enrichment of *CCR5*-disrupted cells using an episomal reporter. (A) Enrichment of GFP^+^ cells after magnetic separation. Scale bar = 50 µm. (B) ZFN-driven mutations detected by the T7E1 assay. Arrows indicate the expected positions of DNA bands cleaved by mismatch-sensitive T7E1. The numbers at the bottom of the gel indicate mutation percentages calculated by band intensities. (C) DNA sequences of the wild-type (WT) and mutant clones, with ZFN recognition sites underlined. Dashes indicate deleted bases, and small bold letters indicate inserted bases. The number of occurrences is shown in parentheses; X1 indicates that each clone was detected once. Mutation frequencies were calculated by dividing the number of mutant clones by the number of total clones.

We first devised reporters that express mRFP, eGFP, and a truncated H-2K^k^ surface marker (H-2K^k^). To allow measurement of the activity of engineered nucleases, we inserted the nuclease target sequence between the sequences encoding mRFP and H-2K^k^ (Figure 1). To prevent the expression of H-2K^k^ and GFP in the absence of the engineered nuclease activity, we prepared double barriers: we made sequences encoding GFP and H-2K^k^ out of frame and also placed a stop codon before GFP and H-2K^k^. When target sequences in the reporter plasmids are cleaved by the engineered nucleases and indels are generated via mutagenic non-homologous end-joining, the frame-shifting mutations generated at the target sequences can make GFP and H-2K^k^ in frame, leading to the expression of GFP and H-2K^k^. To test this reporter system, we cotransfected plasmids encoding the *CCR5*-specific ZFN (Z891) [23] and its reporter into HEK293 cells. CCR5 is a coreceptor of human immunodeficiency virus (HIV) and the knockout of this gene using ZFNs has been reported to prevent HIV infection into T cells [25], [26]. One day after transfection, a significant fraction of cells expressed mRFP, whereas eGFP-expressing cells were hardly observed (Figure S1). The number of eGFP-expressing cells gradually increased over 3 days, suggesting that the ZFN cleaved the target sequence in the reporter plasmid to induce frame-shifting indels [3]. Three days after transfection, H-2K^k^-expressing cells were magnetically separated after labeling with anti-H-2K^k^ antibody conjugated with magnetic beads. Fluorescent microscopy showed that magnetically separated cells were enriched with GFP^+^ cells (Figure 2A). We measured the mutation frequencies (or indel %) in sorted and unsorted cells using T7 endonuclease I (T7E1), an enzyme that specifically recognizes and cleaves heteroduplexes formed by the hybridization of wild-type DNA sequences and mutant sequences. This assay showed that the mutation frequency at the *CCR5* gene in H-2K^k+^ cells was 46%, 12-fold higher than that in unseparated cells (3.7%) (Figure 2B), demonstrating efficient enrichment of *CCR5*-disrupted cells. To confirm this strong enrichment of mutant cells, we next determined the DNA sequences around the target site, and found that the mutation frequency in the magnetically separated cells was 60%, 21-fold higher than that in unseparated cells (Figure 2C). The relatively lower fold enrichment observed with the T7E1 assay as compared to DNA sequencing may be attributable to the fact that at high mutation frequencies, mutant sequences can form homoduplexes, which are insensitive to digestion by T7E1. Thus, the T7E1 assay often underestimates fold enrichments [3].

**Figure 3:**
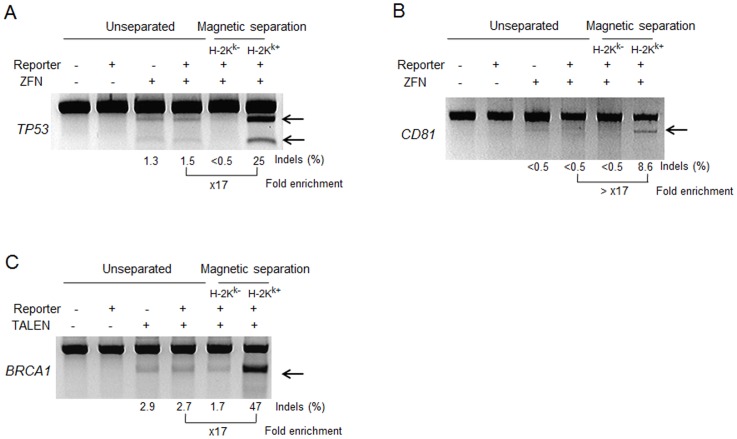
Surrogate reporter-mediated magnetic separations enrich ZFN- and TALEN-driven mutant cells. Nuclease-driven mutations were detected by the T7E1 assay. Arrows indicate the expected positions of DNA bands cleaved by mismatch-sensitive T7E1. The numbers at the bottom of the gels indicate mutation percentages calculated by band intensities.

Next, we tested whether this reporter system is portable to other ZFNs and TALENs. For this, we first used this reporter system with a *TP53* gene-targeting ZFN pair [3] in HEK293 cells. TP53-targeting ZFNs can be used to mutate or repair *TP53*, an important tumor suppressor gene [27]. The T7E1 assay showed that the mutation frequency in magnetically separated cells was 25%, 17-fold higher than that in unseparated cells (1.5%) (Figure 3A). We next tested this reporter using a *CD81*-targeting ZFN pair in a different cell line, Huh 7.5 cells (a human hepatocyte cell line). The T7E1 assay revealed that the mutation frequency was 8.6%, whereas that in the unseparated group was below the detection range (<0.5%) (Figure 3B), suggesting at least 17-fold enrichment of mutant cells. When we performed this reporter-mediated magnetic separation using a *BRCA1*-targeting TALEN pair, the T7E1 assay showed that the mutation frequency in the H-2K^k+^ cells was 47%, 17-fold higher than that in unseparated cells (2.7%) (Figure 3C), suggesting that this magnetic reporter system is compatible with TALENs as well.

### Mutant cell enrichment using hygromycin reporters

**Figure 4:**
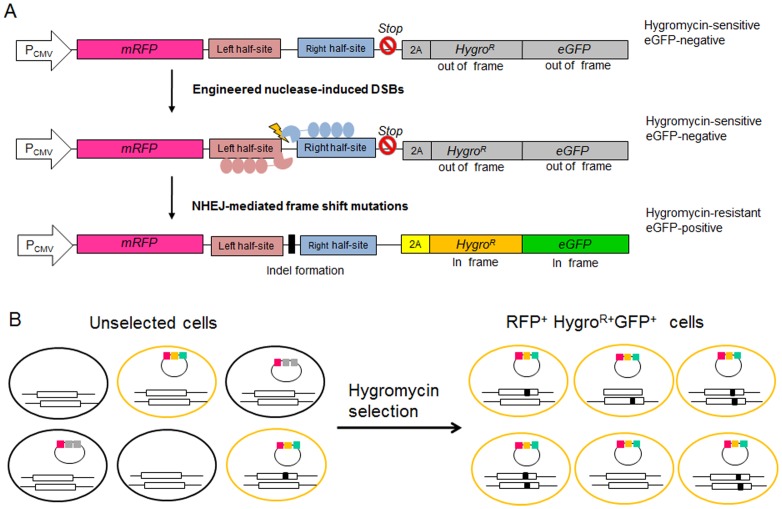
Overview of the episomal reporters used for the enrichment of nuclease-induced mutant cells via hygromycin selection. (A) The working mechanism of the hygromycin reporter. mRFP is constitutively expressed by the CMV promoter (P_CMV_), whereas the *Hygro^R^-eGFP* fusion gene is not expressed in the absence of engineered nucleases because the *Hygro^R^* and *eGFP* sequences are out of frame. If a double-strand break is introduced into the target sequence by engineered nucleases, the break is repaired by non-homologous end-joining (NHEJ), which often results in indels. Indel generation can cause frame shifts, rendering *Hygro^R^-eGFP* in frame and expressed. (B) A schematic depicting the enrichment of mutant cells using the hygromycin reporter. *Hygro^R^-eGFP* fusion gene-expressing cells can be selected using hygromycin treatment. Mutant cells were enriched in this population of *Hygro^R^-eGFP*-expressing cells. Reporter plasmids and chromosomal target loci are shown. Black spots represent mutations.

**Figure 5:**
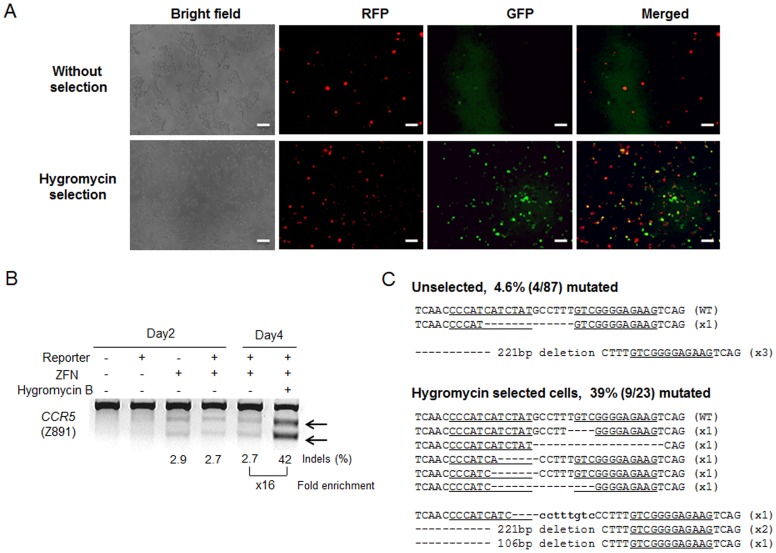
Hygromycin selection through use of a surrogate reporter system enriches nuclease-induced mutant cells. (A) Enrichment of GFP^+^ cells after hygromycin selection. Scale bar = 50 µm. (B) ZFN-driven mutations detected by the T7E1 assay. Arrows indicate the expected positions of DNA bands cleaved by mismatch-sensitive T7E1. The numbers at the bottom of the gel indicate mutation percentages calculated by band intensities. (C) DNA sequences of the wild-type (WT) and mutant clones, with ZFN recognition sites underlined. Dashes indicate deleted bases, and small bold letters indicate inserted bases. The number of occurrences is shown in parentheses; X1, X2, and X3 indicate the number of times that each clone was detected. Mutation frequencies were calculated by dividing the number of mutant clones by the number of total clones.

We next sought to make reporters that rely on neither flow cytometers nor magnetic separation systems. For this, we developed reporters that express a hygromycin-resistance protein (Hygro^R^)-GFP fusion protein only when the target sequences are cleaved by nucleases (Figure 4). Hygromycin treatment after transfection of Z891-encoding plasmids and its reporter into HEK293 cells led to the enrichment of GFP^+^ cells (Figure 5A). The T7E1 assay revealed that the mutation frequency at the *CCR5* gene in the hygromycin-resistant cells was 42%, 16-fold higher than that in unselected cells (Figure 5B). DNA sequencing of this region corroborated this result by showing that the mutation frequency was 39%, 8.5-fold higher than that in unselected cells (4.6%) (Figure 5C). Furthermore, this reporter system allowed 15-fold enrichment of mutant cells induced by a *BRCA1*-targeting TALEN (Figure S2), suggesting that the hygromycin reporters are compatible with TALENs as well as ZFNs.

**Figure 6:**
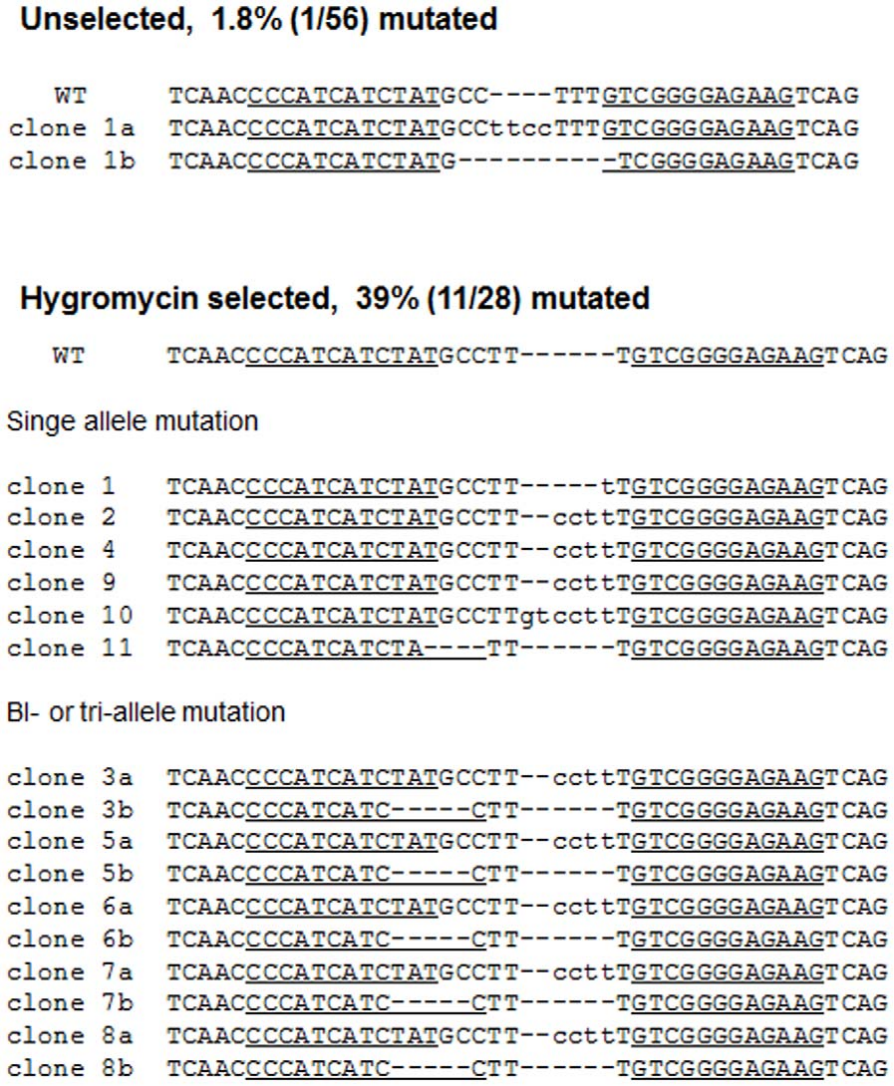
Clonal analysis of hygromycin-selected colonies. After *CCR5*-targeting ZFN (Z891) treatment, the mutant cells were selected by hygromycin treatment. The selected cells were plated at a density of 3,000 cells/100 mm dish, and the clonal colonies were manually picked 10 days after plating. The genomic DNA was isolated from the clonal colonies and analyzed. DNA sequences of the wild-type (WT) and mutant clones are shown, with ZFN recognition sites underlined, deleted bases indicated by dashes, and inserted bases in lower case. The number of occurrences is shown in parentheses; X1 and X2 indicate the number of each clone. Mutation frequencies were calculated by dividing the number of mutant clones by the number of total clones.

We next performed clonal analysis to determine whether hygromycin reporters can facilitate the generation of cells with bi-allelic mutations. After hygromycin treatment, the drug-resistant cells were plated at a density of 3,000 cells/100 mm dish, and the clonal colonies were manually picked 10 days after plating and subjected to analysis. The T7E1 assay revealed that the frequency of mutant colonies in the hygromycin-selected group was 39% (11/28), 22-fold higher than that in the unselected group, in which the frequency was 1.8% (1/56) (Figure S3). Subsequent DNA sequencing confirmed that all 11 colonies were mutant in the hygromycin-selected group, whereas only one colony out of 56 colonies was mutant in the unselected group (Figure 6). Among the 11 colonies, 6 colonies had bi-allelic mutations, suggesting that bi-allelic mutant colonies can be obtained in a highly efficient manner using the hygromycin reporter.

### Comparison of reporters

We next compared the efficiencies of mutant cell enrichment obtained with the two new reporter systems to those obtained via flow cytometry. When a *CCR5*-targeting ZFN pair (Z891) is used, the enrichment of mutant cells using flow cytometric sorting, magnetic separation, and hygromycin selection was 11-, 12-, 16-fold, respectively, suggesting comparable enrichment folds (Table 1). In case of a *TP53*-targeting ZFN pair, the enrichment folds by flow cytometric sorting and magnetic separation were 13- and 17-fold, respectively. Similar fold enrichment was also observed when a *BRCA1*-targeting TALEN pair was used: 17-fold enrichment by magnetic separation and 15-fold enrichment by hygromycin selection. Collectively, enrichment of mutant cells via these new reporter systems was as efficient as that obtained via flow cytometry.

**Table 1:** Efficiencies of mutant cell enrichment via different reporter systems.

Target gene	Mutation frequency (%)		Fold enrichment	Enrichment method	Reference
	Before enrichment	After enrichment			
*CCR5* (Z891 ZFN)	0.8	8.7	11	Flow cytometry	Kim *et al* ^2^
	3.7	46	12	Magnetic separation	This study
	2.7	42	16	Hygromycin selection	This study
*TP53* (ZFN)	2.8	37	13	Flow cytometry	Kim *et al* ^2^
	1.5	25	17	Magnetic separation	This study
*BRCA1* (TALEN)	2.7	47	17	Magnetic separation	This study
	2.3	35	15	Hygromycin selection	This study

We summarized the characteristics of the three reporter systems (Table 2). Hygromycin selection does not need any special instruments or machines, whereas flow cytometric sorting requires flow cytometers, which can be expensive and complicated. Magnetic separation requires magnetic separation instruments, which are much less expensive and simpler than flow cytometers. Thus, if these special facilities or instruments are not available, hygromycin selection would be the choice. If the time required for the enrichment process needs to be short, flow cytometric and magnetic separation would be preferred. These methods take only several hours, whereas hygromycin selection takes several days. Furthermore, hygromycin concentration and exposure time often needs to be determined for each cell type, whereas vigorous optimization processes are less critical in flow cytometric sorting and magnetic separation (although the performance of a flow cytometer machine should be optimized for proper cell sorting). If cells are sensitive to hydrostatic pressure and laser exposure, magnetic separation and hygromycin selection should be considered. Research environments vary and researchers can choose appropriate reporters depending on their experimental conditions.

**Table 2:** Comparison of enrichment methods.

Enrichment method	Flow cytometry	Magnetic separation	Hygromycin selection
**Required machines or instruments**	Flow cytometers	Magnetic instruments	None
**Times required for the enrichment process**	Several hours	Several hours	Several days
**Optimization of the enrichment process**	Usually not necessary (the sorting machine needs optimization)	Usually not necessary	Hygromycin concentration and exposure time need to be optimized for each cell type

In addition, the magnetic and hygromycin reporters can be also used for flow cytometric enrichment of mutant cells because these two reporters express GFP in addition to H-2K^k^ or Hygro^R^ when indels are generated in their target sequences. Thus, our two new reporters will practically replace the previously described fluorescent reporters.

## Conclusions

Here we described two novel episomal reporter systems that can enrich cells with nuclease-induced mutations using magnetic separation and hygromycin selection. The magnetic and hygromycin reporters contain the target sequences of the engineered nucleases and express H-2K^k^ and Hygro^R^, respectively, only when indels are generated in the target sequences by the activity of engineered nucleases. The mutant cell enrichment efficiencies using magnetic and hygromycin reporters were comparable to that using the previously reported fluorescent reporters. Furthermore, our new reporters also allow mutant cell enrichment using flow cytometers as well. Given that ZFNs and TALENs are used in various research environments, our two new reporters will practically replace the previously reported fluorescent reporter system and facilitate the use of engineered nucleases in a wider range of biomedical research.

## Supporting Information

Figure S1 **Expression of RFP and GFP in HEK293 cells after cotransfection of a magnetic reporter plasmid and plasmids encoding a ZFN pair.** HEK293 cells were cotransfected with a magnetic reporter plasmid and plasmids encoding ZFNs that target the *CCR5* gene and observed daily using fluorescent microscopy. Scale bar = 100 µm.
(TIF)

Figure S2 **Enrichment of TALEN-driven mutant cells using the hygromycin reporter.** Two days after a reporter plasmid and plasmids encoding a *BRCA1*-targeting TALEN were cotransfected into HEK293 cells, cells were cultured in either the absence or presence of 2 mg/ml hygromycin for two days. T7E1 assays were performed using genomic DNA isolated from the selected cells. An arrow indicates the expected position of DNA bands cleaved by T7E1.
(TIF)

Figure S3 **Enrichment of clonal populations of cells with ZFN-driven mutations using the hygromycin reporter.** Two days after a reporter plasmid and plasmids encoding ZFN (Z891) were cotransfected into HEK293 cells, hygromycin selection was performed by culturing the cells in the presence of 2 mg/ml hygromycin B for two days. The selected or unselected (control) cells were plated at a density of 3,000 cells/100 mm dish, and the clonal colonies were manually picked 10 days after plating. T7E1 assays were performed using genomic DNA isolated from the colonies. Arrows indicate the expected position of DNA bands cleaved by T7E1. When we analyzed single cell-derived colonies, the frequency of mutant colonies was 39% (11/28) in the hygromycin-selected group and 1.8% (1/56) in the untreated group, demonstrating 26-fold enrichment of mutant cells.
(TIF)

Table S1 **The sequences of primers used in this study.**
(DOCX)

Note S1 **The sequence of the Z891 H-2K^k+^ reporter.** The ZFN recognition site is underlined.
(DOCX)

Note S2 **The sequence of Z891 hygromycin reporter.** The ZFN recognition site is underlined.
(DOCX)
