# Supplementary figures and images for: Magnetic Separation and Antibiotics Selection Enable Enrichment of Cells with ZFN/TALEN-Induced Mutations
Source: PLoS One. 2013 Feb 18;8(2):e56476. doi: 10.1371/journal.pone.0056476 (PMC3575389; doi:10.1371/journal.pone.0056476)

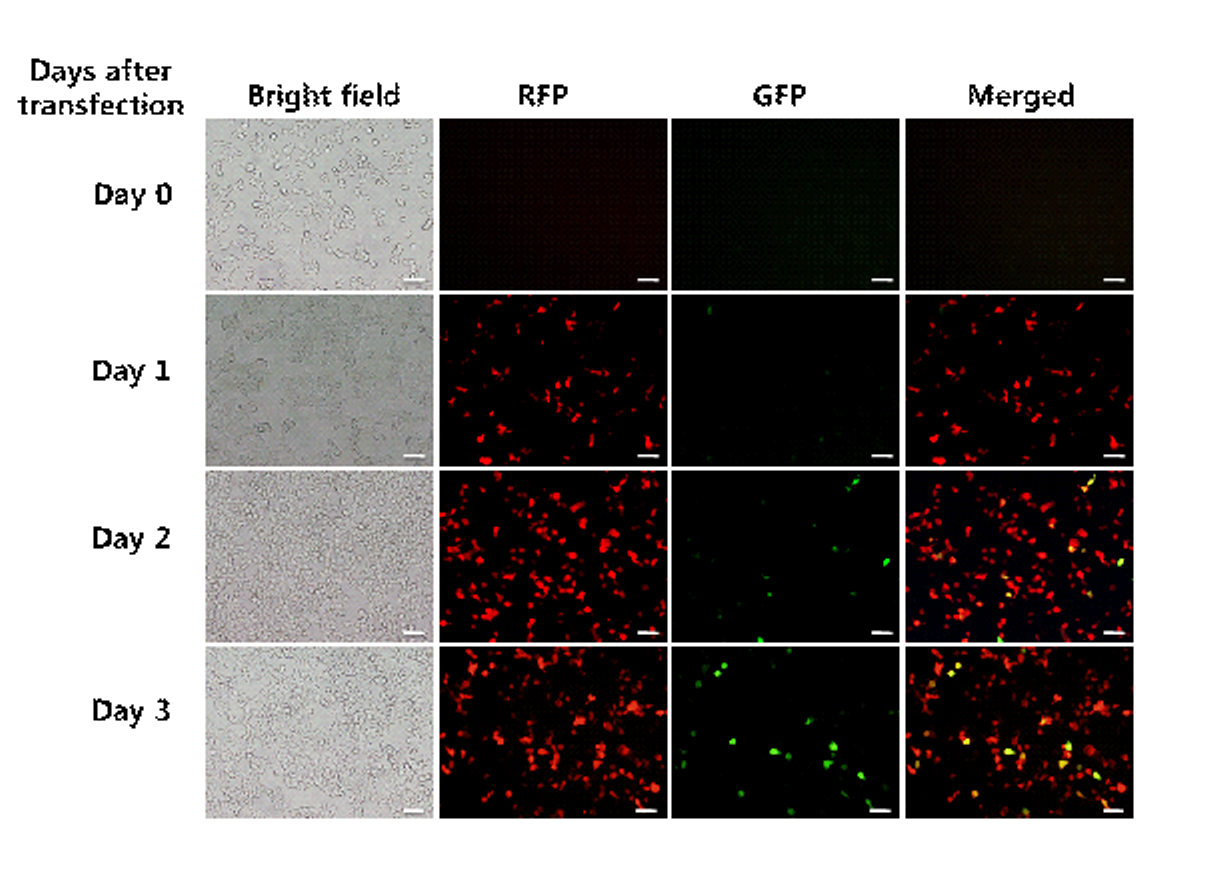

Supplement: Figure S1 — Expression of RFP and GFP in HEK293 cells after cotransfection of a magnetic reporter plasmid and plasmids encoding a ZFN pair. HEK293 cells were cotransfected with a magnetic reporter plasmid and plasmids encoding ZFNs that target the CCR5 gene and observed daily using fluorescent microscopy. Scale bar = 100 µm. (TIF) [file pone.0056476.s001.tif]

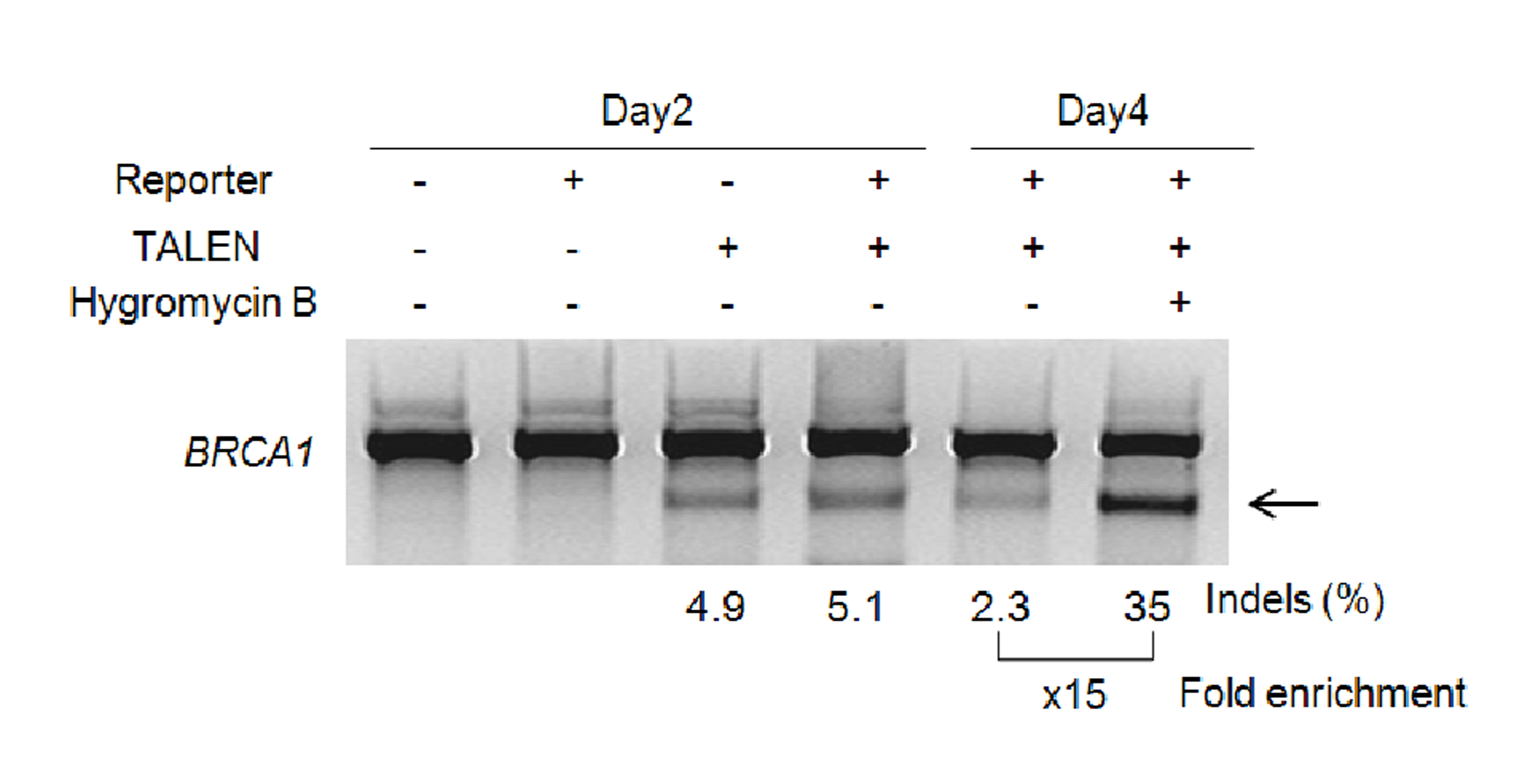

Supplement: Figure S2 — Enrichment of TALEN-driven mutant cells using the hygromycin reporter. Two days after a reporter plasmid and plasmids encoding a BRCA1-targeting TALEN were cotransfected into HEK293 cells, cells were cultured in either the absence or presence of 2 mg/ml hygromycin for two days. T7E1 assays were performed using genomic DNA isolated from the selected cells. An arrow indicates the expected position of DNA bands cleaved by T7E1. (TIF) [file pone.0056476.s002.tif]

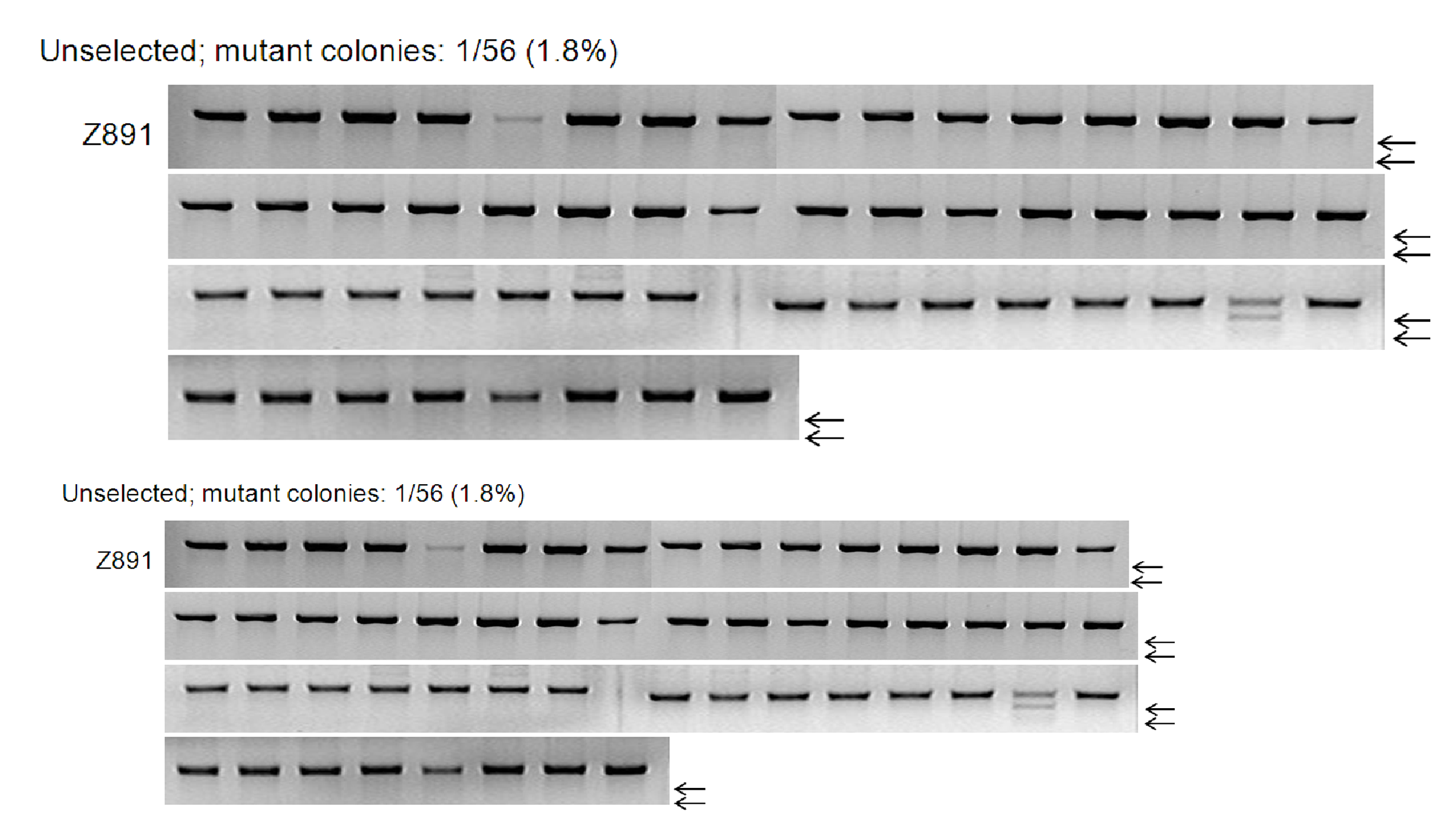

Supplement: Figure S3 — Enrichment of clonal populations of cells with ZFN-driven mutations using the hygromycin reporter. Two days after a reporter plasmid and plasmids encoding ZFN (Z891) were cotransfected into HEK293 cells, hygromycin selection was performed by culturing the cells in the presence of 2 mg/ml hygromycin B for two days. The selected or unselected (control) cells were plated at a density of 3,000 cells/100 mm dish, and the clonal colonies were manually picked 10 days after plating. T7E1 assays were performed using genomic DNA isolated from the colonies. Arrows indicate the expected position of DNA bands cleaved by T7E1. When we analyzed single cell-derived colonies, the frequency of mutant colonies was 39% (11/28) in the hygromycin-selected group and 1.8% (1/56) in the untreated group, demonstrating 26-fold enrichment of mutant cells. (TIF) [file pone.0056476.s003.tif]
