# Supplementary material for: Magnetic Separation and Antibiotics Selection Enable Enrichment of Cells with ZFN/TALEN-Induced Mutations
Source: PLoS One. 2013 Feb 18;8(2):e56476. doi: 10.1371/journal.pone.0056476 (PMC3575389; doi:10.1371/journal.pone.0056476)
Supplement: Table S1 — The sequences of primers used in this study. (DOCX) [file pone.0056476.s004.docx]

**Primers for reporter construction**

| ***vH2-k^k^*** | F | 5'-gagtttcaacaaagcgtagttagtacattgcttgtacagctcgtccatgc-3' |
| --- | --- | --- |
| ***vH2-k^k^*** | R | 5’-agaaggagaaacacaggtggaaaaggagggtaaagcggccgcgactctag-3' |
| ***iH2-k^k^*** | F | 5'-atcactctcggcatggacgagctgtacaagcaatgtactaactacgcttt-3' |
| ***iH2-k^k^*** | R | 5’-tgattatgatctagagtcgcggccgctttaccctcctttt-3' |
| ***Hygro*** | F | 5'-ggcgctagcatgaaaaagcctgaactcaccg-3' |
| ***Hygro*** | R | 5-gcgtctagagtcgacttcctttgccctcggacgagtg-3' |
