# Supplementary material for: Magnetic Separation and Antibiotics Selection Enable Enrichment of Cells with ZFN/TALEN-Induced Mutations
Source: PLoS One. 2013 Feb 18;8(2):e56476. doi: 10.1371/journal.pone.0056476 (PMC3575389; doi:10.1371/journal.pone.0056476)
Supplement: Note S1 — The sequence of the Z891 H-2Kk+ reporter. The ZFN recognition site is underlined. (DOCX) [file pone.0056476.s005.docx]

atggcctcctccgaggacgtcatcaaggagttcatgcgcttcaaggtgcgcatggagggctccgtgaacggccacgagttcgagatcgagggcgagggcgagggccgcccctacgagggcacccagaccgccaagctgaaggtgaccaagggcggccccctgcccttcgcctgggacatcctgtcccctcagttccagtacggctccaaggcctacgtgaagcaccccgccgacatccccgactacttgaagctgtccttccccgagggcttcaagtgggagcgcgtgatgaacttcgaggacggcggcgtggtgaccgtgacccaggactcctccctgcaggacggcgagttcatctacaaggtgaagctgcgcggcaccaacttcccctccgacggccccgtaatgcagaagaagaccatgggctgggaggcctccaccgagcggatgtaccccgaggacggcgccctgaagggcgagatcaagatgaggctgaagctgaaggacggcggccactacgacgccgaggtcaagaccacctacatggccaagaagcccgtgcagctgcccggcgcctacaagaccgacatcaagctggacatcacctcccacaacgaggactacaccatcgtggaacagtacgagcgcgccgagggccgccactccaccggcgccgaattc***cccatcatctatctcgaggtcggggagaag***tgaaggatccagtgagcaagggcgaggagctgttcaccggggtggtgcccatcctggtcgagctggacggcgacgtaaacggccacaagttcagcgtgtccggcgagggcgagggcgatgccacctacggcaagctgaccctgaagttcatctgcaccaccggcaagctgcccgtgccctggcccaccctcgtgaccaccctgacctacggcgtgcagtgcttcagccgctaccccgaccacatgaagcagcacgacttcttcaagtccgccatgcccgaaggctacgtccaggagcgcaccatcttcttcaaggacgacggcaactacaagacccgcgccgaggtgaagttcgagggcgacaccctggtgaaccgcatcgagctgaagggcatcgacttcaaggaggacggcaacatcctggggcacaagctggagtacaactacaacagccacaacgtctatatcatggccgacaagcagaagaacggcatcaaggtgaacttcaagatccgccacaacatcgaggacggcagcgtgcagctcgccgaccactaccagcagaacacccccatcggcgacggccccgtgctgctgcccgacaaccactacctgagcacccagtccgccctgagcaaagaccccaacgagaagcgcgatcacatggtcctgctggagttcgtgaccgccgccgggatcactctcggcatggacgagctgtacaagcaatgtactaactacgctttgttgaaactcgctggcgatgttgaaagtaaccccggtcctgctagcatggcaccctgcatgctgctcctgctgttggcggccgccctggccccgactcagacccgcgcgggcccacattcgctgaggtatttccacaccgccgtgtcccggcccggcctcgggaagccccggttcatctctgtcggctacgtggacgacacgcagttcgtgcgcttcgacagcgacgcggagaatccgaggtatgagccgcgggtgcggtggatggagcaggtggagcccgagtattgggagcggaacacgcagatcgccaagggcaatgagcagattttccgagtgaacctgaggaccgcgctgcgctactacaaccagagcgcgggcggctctcacacgttccaacggatgtacggctgtgaggtggggtcggactggcgcctcctccgcgggtacgagcagtacgcatacgacggctgcgattacatcgccctgaacgaagacctgaaaacgtggacggcggccgacatggcggcgctgatcaccaaacacaagtgggagcaggctggtgatgcagagagagaccgggcctacctggagggcacgtgcgtggagtggctccgcagatacctgcagctcgggaacgcgacgctgccgcgcacagattccccaaaggcccatgtgacccgtcacagcagacctgaagataaagtcaccctgaggtgctgggccctgggcttctaccctgctgacatcaccctgacctggcagttgaatggggaggagctgacccaggacatggagcttgtggagaccaggcctgcaggggatggaaccttccagaagtgggcatctgtggtggtgcctcttgggaaggagcagtattacacatgccatgtgtaccatcaggggctgcctgagcccctcaccctgagatgggagcctcctccatccactgtctccaacacggtaatcattgctgttctggttgtccttggagctgcaatagtcactggagctgtggtggcttttgtgatgaagatgagaaggagaaacacaggtggaaaaggagggtaa
